# P-153. Malaria in a military hospital in Bogotá, Colombia, 2023 – 2024

**DOI:** 10.1093/ofid/ofae631.358

**Published:** 2025-01-29

**Authors:** Leidy J Medina-Lozano, Julián Camilo Oviedo Naranjo, Mariana espinosa Murcia, Álvaro A Faccini-Martínez

**Affiliations:** Hospital Militar Central, Bogotá, Distrito Capital de Bogota, Colombia; Universidad Militar Nueva Granada, Bogotá, Distrito Capital de Bogota, Colombia; Universidad Militar Nueva Granada, Bogotá, Distrito Capital de Bogota, Colombia; Hospital Militar Central, Bogotá, Distrito Capital de Bogota, Colombia

## Abstract

**Background:**

In Colombia malaria is an endemic mosquito-borne disease. Although military personnel are recognized as a high-risk group for malaria, little is known about on features of this parasitic disease in Colombian military members.

**Figure:**
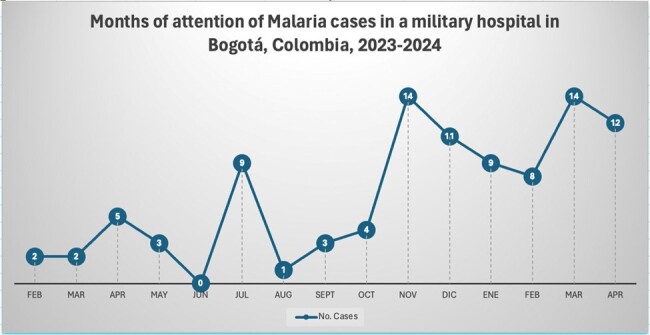

**Methods:**

We recorded data on demographic, clinical, laboratory and treatment of patients with confirmed malaria diagnosis (positive thick blood smear or Rapid Diagnostic Test), attended in a reference military hospital, located in Bogotá, Colombia, from February 2023 to April 2024. Cases of severe malaria and recurrence were classified following the last WHO guidelines. Dengue and malaria coinfection were defined as a confirmed malaria case with positive dengue NS1 antigen.

**Figure:**
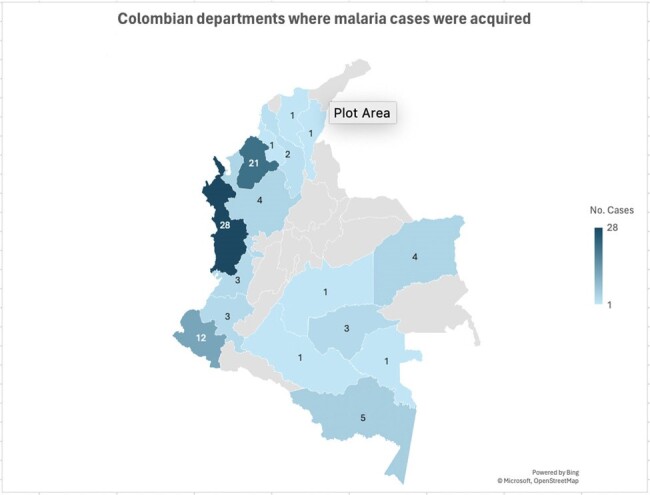

**Results:**

During the study period, 97 patients were attended; all of them males, in active military service, and the median age was 25 years. Most (70%) consulted between November 2023 and April 2024 (Figure 1), and the main Colombian departments where the infections were acquired were Chocó (29%), Córdoba (22%) and Nariño (12%) (Figure 2). *Plasmodium vivax* was the main etiology (79%), follow by *P. falciparum* (17%), mixed infections (3%) and *Plasmodium* sp. (1%); four patients had coinfection with dengue. Most (51%) were attended at the emergency department, 39% at general ward, and 10% required intensive care unit. Recurrences were diagnosed in 36%, and the remaining as new infections. Among clinical features, the main symptoms were fever (88%), headache (75%) and chills (64%); and the main signs were jaundice (25%), abdominal pain (21%) and mild dehydration (12%) (Table 1). The median days of fever at hospital admission was five. Severe malaria was diagnosed in 8 patients. Among laboratory findings, thrombocytopenia was the most frequent (77%), follow by hyperbilirubinemia (71%). Hepatosplenomegaly was the most finding at the abdominal ultrasound (49%) (Table 2). Regarding treatment, 92% of *P. vivax* cases received chloroquine-primaquine; 69% of *P. falciparum* cases received artemether-lumefantrine and single dose primaquine; mixed infections cases received artemether-lumefantrine and primaquine; and all severe malaria received intravenous artesunate. No deaths were recorded.

**Figure:**
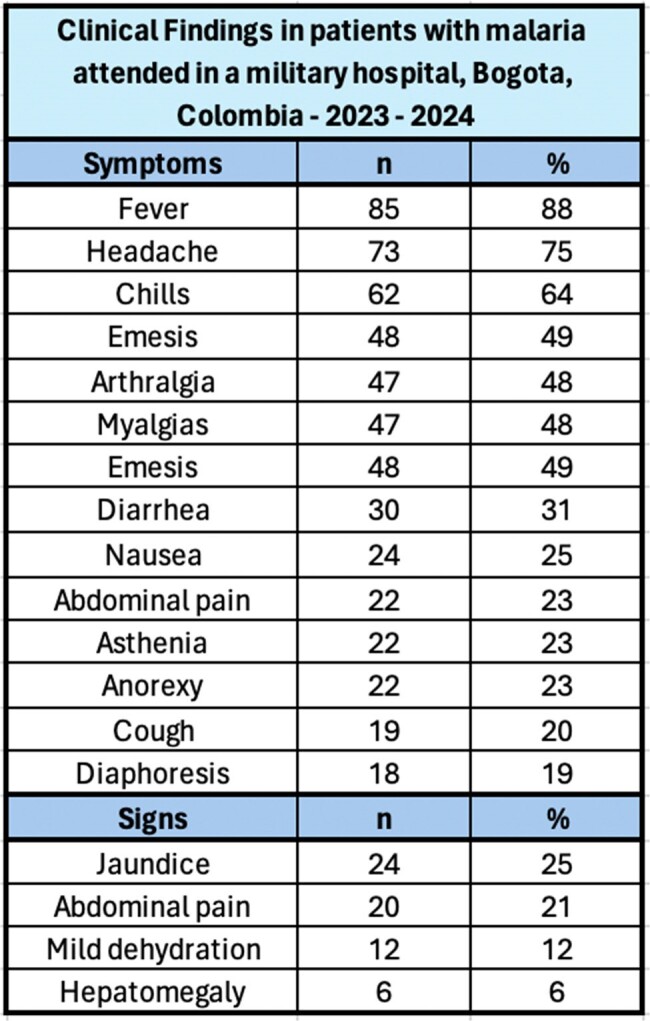

**Conclusion:**

Malaria remains a potential health threat to Colombian military personnel.

**Figure:**
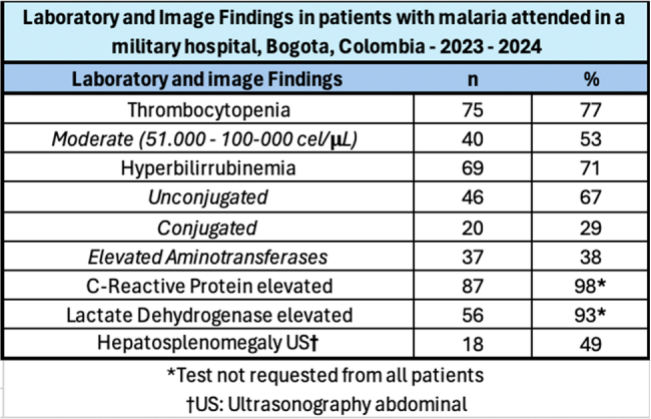

**Disclosures:**

**All Authors**: No reported disclosures

